# Language Development From Early Childhood to Adolescence in Youths With Fragile X Syndrome

**DOI:** 10.1044/2020_JSLHR-20-00198

**Published:** 2020-10-01

**Authors:** Nancy C. Brady, Kandace Fleming, Shelley L. Bredin-Oja, Heather Fielding-Gebhardt, Steven F. Warren

**Affiliations:** a Department of Speech-Language-Hearing: Sciences & Disorders, The University of Kansas, Lawrence; b Life Span Institute, The University of Kansas, Lawrence

## Abstract

**Purpose:**

The aim of this study was to investigate language growth in individuals with fragile X syndrome (FXS) from early childhood to adolescence and the influence of maternal responsivity on language growth.

**Method:**

We conducted a longitudinal analysis of language development in 55 youths (44 males, 11 females) with FXS. Data collection spanned the ages of 11–216 months. We measured expressive and receptive vocabulary with standardized tests. The number of different words and mean length of utterance were obtained from language sample analyses of mother–child interactions. We also measured maternal comments (responsivity indicator) produced during the language samples and child nonverbal IQ.

**Results:**

Growth models indicated that rates of number of different words and receptive vocabulary were related to maternal commenting. Mean length of utterance did not change significantly over time. Expressive vocabulary measured with a standardized test grew, but the growth was not related to maternal commenting. Nonverbal IQ was related to all language outcomes at age of 10 years and to changes over time in vocabulary. Visual analysis indicated that the highest scores on standardized tests were produced by girls; however, measures derived from language sample analyses appeared similar for boys and girls. Language models for boys only were similar to the total sample models with lower scores at age of 10 years for some outcomes.

**Conclusion:**

Results of persistent language impairments for most youths with FXS suggest the need for continued, focused interventions aimed at improved language productions in addition to a responsive environment.

**Supplemental Material:**

https://doi.org/10.23641/asha.13022825

Fragile X syndrome (FXS) is an X-linked disorder associated with delays in cognitive and language development, particularly in males. This inherited disorder affects approximately 1.4 per 10,000 males and 0.9 per 10,000 females (Hunter et al., 2014). Our longitudinal research project has been documenting the developmental trajectories of communication and language in a group of 55 children with FXS, with an emphasis on how maternal responsivity relates to differences in these trajectories (Brady et al., 2014; Warren et al., 2010). The purpose of this study is to describe how maternal responsivity is related to growth in language development from childhood into adolescence.

## Language Development in FXS

Most males and many females with FXS have impaired communication (Abbeduto et al., 2016). Impairments have been reported in the areas of speech intelligibility (Barnes et al., 2009), vocabulary (Roberts et al., 2007; Sudhalter & Belser, 2001), morphology and syntax (Komesidou et al., 2017; Martin et al., 2013), and pragmatics (Martin et al., 2018). In addition, individuals with FXS have impaired cognition, and there is a relationship between the amount of impairments in cognition and communication (Abbeduto et al., 2007; Oakes et al., 2013).

A subset of individuals with FXS also meet diagnostic criteria for autism, and those with autism have been reported to have poorer language outcomes (Haebig et al., 2020; Martin et al., 2012). However, individuals who meet diagnostic criteria for autism also tend to have more impaired cognition (Haebig et al., 2020; Klusek et al., 2014), and it can be difficult to determine if language outcomes are more affected by IQ, autism status, or some combination of these two variables (Thurman et al., 2017, 2015). In previous studies by our research team, we have found cognition and autism to be closely related to each other and to language outcomes (Warren et al., 2010).

### Maternal Responsivity and Language Development in FXS

A long line of research has demonstrated that the way parents interact with their children can impact language development. For example, responding contingently to children's communication attempts has been associated with improved language outcomes for participants between the ages of 2 and 12 years learning to use augmentative and alternative communication (O'Neill et al., 2018). Conversely, 3-year-old children whose parents did not respond positively to their communication at age of 2 years have been found to have lower language levels (Hirsh-Pasek et al., 2015).

Our research has focused on how maternal responsivity relates to language outcomes for children with FXS during early (Warren et al., 2010) and middle (Brady et al., 2014) childhood. Our longitudinal study began when children were between 11 and 48 months of age (*M* = 28.2 months). We collected data on mother–child interactions and child language attainments approximately once every 18 months until children were between 9 and 10 years old. Using growth curve modeling, we showed that early and sustained maternal responsivity predicted child language outcomes. Specifically, relatively high rates of maternal responsivity during mother–child interactions were predictive of many language variables, including rates of communication and receptive and expressive vocabulary scores on the Mullen Scales of Early Learning (Brady et al., 2014; Hahn et al., 2014; Mullen, 1995; Warren et al., 2010).

Komesidou et al. (2017) examined development of syntax over time, as measured by the Index of Productive Syntax (IPSyn; Scarborough, 1990). Language samples collected at each observation were analyzed to measure morphological and syntactic structures and mean length of utterance (MLU) in words. Both IPSyn scores and MLU in words grew over time, but at much slower rates than in typically developing (TD) children. Child IQ and autism spectrum disorder (ASD) symptoms predicted variance in growth of these measures; however, maternal responsivity was not predictive. That is, children with more responsive mothers did not appear to have a general advantage in syntactic development.

Taken together, these findings suggest that maternal responsivity is a significant predictor of variability in specific components of language, such as vocabulary and overall communication rates in children with FXS. Although syntax was not predicted by responsivity in the study by Komesidou et al. (2017), it is possible that these results were affected by the limited development in syntax among the subjects. That is, many children were still in the process of acquiring basic syntax and had insufficient numbers of verbal utterances to analyze. One purpose of the current study is to further consider how responsivity may relate to language outcomes in adolescents with FXS, many of whom had more complex language than at earlier ages.

### Adolescent Language in FXS

Results of standardized language tests completed with male adolescents with FXS have shown impairments in multiple areas of receptive and expressive language (Roberts et al., 2007; Thurman et al., 2017). Analyses of language samples obtained during narration and conversation also revealed impairments in vocabulary and syntax relative to typical development (Abbeduto et al., 2016). Abbeduto et al. (2016) noted that some boys with FXS remain essentially nonverbal as they enter adolescence.

For individuals who progress in spoken language, deficits in vocabulary and grammatical productions have been identified (Kover & Abbeduto, 2010), but these deficits are largely commensurate with other areas of development. In comparison to TD age-matched children, adolescents with FXS had similar outcomes for MLU, percentage of grammatical communication units, clause density, and mean number of causal conditional connectors obtained from a narrative task (Keller-Bell & Abbeduto, 2007). Similarly, when grammatical construction was measured with the Developmental Sentence Scoring system (Lee, 1974), Finestack and Abbeduto (2010) found that male adolescents with FXS had similar or better total Developmental Sentence Scoring scores than TD children matched on nonverbal mental age. However, it should be noted that Finestack and Abbeduto only included adolescents with FXS who had relatively high levels of expressive language abilities (MLU above 3.0) in their analyses.

A number of studies have compared expressive language production in adolescents with FXS to other groups who have intellectual and developmental disabilities. Levy et al. (2006) found that adolescents with FXS were less impaired than comparison groups who had comorbid FXS and ASD or Down syndrome. However, it should be noted that language in participants with FXS was consistently below chronological age expectations. In a study comparing grammatical productions by young adolescents with FXS (*M*_age_ = 12.12) or ASD (*M*_age_ = 13.4), Sterling (2018) reported better grammatical productions by participants with FXS in comparison to participants with FXS and ASD, even though they had similar MLUs (calculated in morphemes). However, as in the Finestack and Abbeduto (2010) study, the average MLUs in Sterling's study were also above 3.0, and it is not clear if similar results would be obtained across individuals presenting with a broader range of language skills.

A study by Martin et al. (2013) examined language growth in children with FXS between the ages of 10 and 13 years and compared this growth to children with FXS plus ASD, boys with Down syndrome, and a TD group matched according to nonverbal mental age. The TD children were approximately 5 years of age at the start of the study and 7 years of age at the conclusion. Participants' expressive vocabulary, syntax, and pragmatic language skills were assessed with the Comprehensive Assessment of Spoken Language (Carrow-Woolfolk, 1999). Results showed that the participants with FXS only and FXS plus ASD had significantly lower age-equivalent scores at the initial observation and had significantly shallower slopes over time compared to the TD participants. In other words, the language outcomes were lower than expected for nonverbal mental age when participants with FXS were 10 years of age, and the deficit relative to the TD children continued to grow over the next 3 years.

The boys with FXS in the study by Martin et al. (2013) did have positive trajectories, however, indicating growth. The reported ranges for each subtest of the Comprehensive Assessment of Spoken Language indicated considerable variability across the boys with FXS. For example, means for the Syntax Construction subtest ranged between 2.1 and 6.3 at Visit 1; at Visit 3, they were between 2.2 and 9.0. Thus, it appears that some participants changed much more than others. Martin et al. (2013) found that ASD severity was predictive of outcomes in the Pragmatic Judgment subscale but not for other domains, including syntax.

## Current Investigation

There is a need for additional longitudinal studies to examine language development in individuals with FXS, particularly as observed during natural interactions (i.e., language samples). As summarized previously, results from standardized tests may differ from language produced during actual conversations. In addition, there is a need to begin longitudinal studies at earlier stages of language development to identify predictors of different outcomes and to be able to better predict the course of language development for individual children (Finestack & Abbeduto, 2010).

The current investigation provides an opportunity to study aspects of language development from early childhood to adolescence in children with FXS. Longitudinal studies that span this length of development face numerous challenges. One such challenge is the selection of measures. Measures that are ideal for characterizing communication and language at young ages may no longer be valid or appropriate for older children and adolescents. Standardized tests contain items that were standardized for specific ages, and these items may not be appropriate outside specified age ranges. Although language sampling does not have limitations associated with standardization, the appropriateness of contexts used for eliciting language samples may change over time and thus affect results. For example, joint book reading is a context often used to investigate language skills with young children, but adolescents may be substantially less likely to engage in joint book reading with parents. Hence, the sample may not be representative, and validity may be compromised.

In addition to measurement contexts, the indicators of constructs change over time. For example, in the current study, we are examining how maternal responsivity relates to language growth. However, the behaviors that are considered responsive during early childhood may not be the same as those observed during adolescence. We found that a composite of maternal commenting, requests for verbalizations (e.g., What is that?), and recoding of child communication attempts were factors contributing to the construct of responsivity during early childhood (Warren et al., 2010). However, as described under the Measures section, these same behaviors were not all indicators of responsivity during adolescence—only comments continued to reflect a responsive parenting style. Therefore, in the current study, we investigated the role of maternal commenting on language growth in individuals with FXS from early childhood to adolescence. Our specific research questions were as follows:

1. What are the trajectories for the following language outcomes measured from early childhood to adolescence in participants with FXS: receptive vocabulary, expressive vocabulary, rate of different words, and MLU in morphemes (MLU_m_)?
2. What is the trajectory of maternal commenting—our index of responsivity—over this same time period?
3. Does the commenting trajectory predict trajectories of receptive vocabulary, expressive vocabulary, rate of different words, and MLU_m_ controlling for the effect of cognition on language outcomes?

## Method

Our research was approved by the Human Subjects Committee of the University of Kansas. The approval number is CR00008915. All families provided informed consent and assent before participating.

### Participants

Fifty-five children with FXS (11 girls) and their mothers participated in an ongoing longitudinal study (Brady et al., 2014; Warren et al., 2010). For the current study, data were collected across as many as 7 data points, extending from toddlerhood into adolescence. The study design called for data collection every 18 months during childhood. The average time between childhood data collection periods was 19.06 months. Data collection began again during adolescence, and the average time between the last childhood visit and the first adolescent visit was 76.91 months. Data were collected from each child between 3 and 7 times (*M* = 6), with 64% of participants contributing 6 data points, 18% contributing 7 data points, 15% contributing 5 data points, 2% contributing 4 data points, and 2% contributing 3 data points. Forty-three of the 55 participants were observed during the adolescent period. The participants ranged in age from 11 to 48 months at their first observation, with a mean of 29 months (*SD* = 8.8). For the 43 participants observed in the adolescent period, age ranged from 13;4 to 18;0 (years;months), with a mean of 15;11 (*SD* = 1;2). Twelve participants from the original cohort did not provide data for the adolescent period because we either had not yet completed a measure during the adolescent period (*n* = 3) or were unable to contact them during the adolescent period (*n* = 9).

Mother and child genotypes were confirmed through blood sample analyses. Forty-nine mothers carried the *FMR1* premutation, two mothers were mosaic for premutation and full mutation, and three carried the full mutation. At the beginning of the study, mothers ranged in age from 22.33 to 39.77 years, and at the adolescent period, mothers ranged in age from 36.72 to 55.07 years. All child participants had full mutation FXS. Child participants demonstrated substantial variances in cognition, language, and adaptive behaviors. Their skills at earlier ages are summarized in previous publications (e.g., Brady et al., 2014; Warren et al., 2010), and skills through adolescence are presented in the Results section.

Participating families were recruited through FXS family support group networks, advertising at national conventions, an FXS parent e-mail list, and through a national research registry at the University of North Carolina at Chapel Hill. Given that FXS is a rare disorder, the participants represent a sample of convenience. However, there was some diversity on demographic factors (see Table 1).

**Table 1.** Demographic information for study participants.

Demographic factor	*n*	Level	Frequency	%
Child ethnicity	54	Hispanic	2	3.7
		Non-Hispanic	52	96.3
Mother ethnicity	54	Hispanic	2	3.7
		Non-Hispanic	52	96.3
Child race^a^	54	Native American	—	—
		Asian	1	1.8
		Native Hawaiian/Pacific Islander	1	1.8
		Black	6	11.1
		White	50	92.6
Mother race^a^	54	Native American	1	1.8
		Asian	1	1.8
		Native Hawaiian/Pacific Islander	1	1.8
		Black	3	5.6
		White	49	90.7
Mother's education	51	High school or GED	8	15.7
		Some post high school	11	21.6
		Bachelor's degree	18	35.3
		Post bachelor's or advanced degree	14	27.5
Household income	54	< $15,000	3	5.6
		$15,000–$29,999	7	13.0
		$30,000–$49,999	7	13.0
		$50,000–$79,999	10	18.5
		$80,000–$99,999	8	14.8
		> $100,000	18	33.3
Child methylation status	55	Fully methylated	46	83.6
		Partially methylated	9	16.4
Communication modalities^b^	55	Signs	8	14.5
		Verbalization	44	80.0
		AAC	1	1.8

*Note.* GED = General Educational Development; AAC = augmentative and alternative communication.

a Participants could indicate one or more races.

b Modality was used at least once during parent–child interaction.

### Measures

#### Language Outcome Measures

We obtained the children's number of different words and MLU_m_ from a language sample collected during a mother–child interaction. We provided each mother–child dyad with materials to make and eat a snack together. The length of this activity was 5 min during the toddler and childhood periods and 10 min for the adolescent period; therefore, rate per minute was used for all analyses. It should be noted that, although other contexts were recorded at each observation, some of the contexts changed over time to reflect age-appropriate interests. For example, during the first few observations, dyads were given materials to make a craft together, whereas at later observations, this activity was replaced with an interactive three-dimensional puzzle. We identified some differences associated with these contexts and hence were limited in our analysis to the one consistent context over time—snack.

Child utterances during the snack recordings were transcribed from the digital videotapes using the Noldus Observational Coding system (Noldus Information Technology, 2002). Words produced in any modality (i.e., speech, sign, and augmentative and alternative communication) were included, although the vast majority of words were spoken. All files were initially transcribed by a primary coder. A secondary coder checked the transcript and marked utterances for disagreements. All changes to the original transcript were resolved by consensus. Disagreements that could not be resolved were marked as unintelligible. The transcripts were analyzed using the Systematic Analysis of Language Transcripts (SALT; 2016) software. Research lab personnel who were trained in SALT procedures separated the utterances into communication units, defined as a main clause and all of its subordinate clauses. They also marked all bound morphemes according to the SALT manual (Miller & Iglesias, 2015). We used the number of different words and MLU_m_ as dependent measures in our analyses. For the MLU_m_, we limited the analysis to samples that contained at least 10 verbal utterances. Although this is a small number of utterances, Casby (2011) found MLUs were similar and not statistically different when obtained from samples of 10 or 100 utterances.

We also administered two standardized vocabulary tests, the Expressive Vocabulary Test–Second Edition (EVT-2; Williams, 2007) and the Peabody Picture Vocabulary Test–Fourth Edition (PPVT-4; Dunn & Dunn, 2007), at each observation. The EVT-2 is a standardized test that measures expressive vocabulary and word retrieval for Standard American English. The PPVT-4 measures receptive vocabulary using a picture identification task. For both the EVT-2 and the PPVT-4, we used the raw scores for the total number of correct responses at each administration in our analyses, as this is more reflective of growth over time than standard scores (Hessl et al., 2009; Sterling & Abbeduto, 2012). These two standardized assessments of language have been widely used to characterize language ability in individuals with FXS (del Hoyo Soriano et al., 2018; Haebig & Sterling, 2017; Haebig et al., 2020; Martin et al., 2017; McDuffie et al., 2012; Roberts et al., 2007). The EVT-2 has very high split-half and alpha reliabilities, suggesting strong internal consistency and reliability. The split-half reliability ranges from .88 to .97 depending on age, and the alpha reliability ranges from .94 to .98 depending on age. Similarly, the PPVT-4 also has very high internal consistency and reliability. The split-half reliability ranges from .89 to .97 depending on age, and the alpha reliability ranges from .93 to .98.

#### Nonverbal IQ

The Leiter International Performance Scale–Revised (Leiter-R; Roid & Miller, 1997) was used to assess nonverbal cognitive ability at each middle childhood and adolescent observation. Participants completed the Brief IQ screener, which consists of the Figure Ground, Form Completion, Sequential Ordering, and Repeated Patterns subtests. This assessment has been widely used to measure nonverbal IQ in individuals with FXS (Haebig et al., 2020; Klusek et al., 2014; McDuffie et al., 2010, 2015). The Leiter-R has acceptable internal consistency. Specifically, the average alpha reliability coefficients for the Figure Ground, Form Completion, Sequential Ordering, and Repeated Patterns subtests are .75, .88, .75, and .76, respectively. For purposes of analysis, the score from their observation at age of 10 years was used as a covariate. The children averaged 9.5 years at the time of the assessment (*SD* = 3.5 months, range: 8.4–10.1 years). The Nonverbal IQs had a mean of 52, so scores were centered there to facilitate interpretation of the other variables in the model. Nonverbal IQs ranged from 36 to 91, with an *SD* of 13.8.

#### Maternal Responsivity

As explained in the introduction, a primary goal of this study was to further investigate the role of maternal responsivity in predicting language growth in children with FXS through adolescence. In past studies (Brady et al., 2014; Warren et al., 2010), we used a composite score that comprised multiple indicators of responsivity: comments, requests for verbal complies, and recodes. However, the value and appropriateness of these maternal communication behaviors may change over time. For example, once a child is talking, it may no longer be facilitative for the mother to request a verbal compliance (e.g., “Tell me what this is” while pointing to a picture). Similarly, recoding a child's communication attempt (e.g., child says “ba,” and mother says “ball”) makes sense and is likely helpful when the child is gesturing or producing difficult-to-understand speech (Saxton, 2005). However, recoding a completely intelligible verbal utterance is not common in typical conversations and may even be disruptive to the flow of conversation. Because of these concerns, we decided to use maternal comments as our indicator of responsivity in this study.

Comments were defined as all verbal comments to the child, for example, saying “Good job!” or “I'm going to the store.” Comments exclude requests for verbal compliance (e.g., “Tell me what you want”) and requests for behavioral compliance (e.g., “Please sit down”). In the current study, we examined maternal comments during snack as a time-varying predictor. This allowed us to determine how differences in commenting within a person over time and differences in commenting between people related to the trajectories of language outcomes.

*Fidelity of coding.* All utterances produced by the mother were transcribed from the videotaped interactions by a trained primary coder using the Noldus Observational Coding system (Noldus Information Technology, 2002) and coded as either a comment, a request for a verbal response from the child, or a request for a behavior from the child. A second coder, who was also trained, watched the video and reviewed the transcript and codes. Any disagreements were noted on a spreadsheet. The two coders resolved differences by consensus; if a consensus could not be reached, a third trained coder in the lab was consulted. Through this procedure, all files were coded by consensus by at least two trained coders.

### Data Analysis

General linear mixed models with repeated observations nested within persons were used to examine maternal commenting as a predictor of age-related change in child communication. Specifically, multivariate longitudinal models were used to jointly examine the trajectories of rate of maternal commenting and child communication outcomes. Within the models, we appropriately examined both the between-dyads and within-dyad sources of variation. The between-dyads parameters examined the extent to which some mothers commented more than others and the extent to which some children communicated more than others. The within-dyad parameters examined the extent to which a mother commented more or less at a given occasion relative to her own rate of commenting over time and the extent to which a child communicated more or less at a given occasion relative to their communication over time. Nonverbal IQ was added to each model so that associations with language outcome growth parameters could be examined. This also meant that the examination of the role of maternal responsivity on language outcomes controlled for established associations between nonverbal IQ and language.

Four separate models, one for each child language outcome (i.e., rate of different words, EVT-2 raw scores, PPVT-4 raw scores, and MLU_m_) were evaluated. Sample sizes and the number of observations available varied across outcomes, which had implications for the types of growth models used as described in the results for each outcome. We examined the predictive influence of nonverbal IQ and maternal commenting for each of these outcomes. Fixed intercepts and slopes were used to represent the mean level and rate of linear age-related change across individuals in the sample for both maternal commenting and for child outcomes. To represent individual differences in trajectories (i.e., within-dyad) for the maternal commenting predictor and child outcomes, random intercepts (each person's deviation from the fixed intercept) and random linear slopes (each person's deviation from the fixed slope) were evaluated for each dyad on each variable. Additionally, a residual (the difference between predicted individual scores based on intercept and slope and actual observed scores for the individual) was added to the models for each outcome. Because we were interested in associations among the growth parameters across variables, we retained random effects in the models if the effect was significant for either of the outcomes being modeled rather than finding the best-fitting Level 1 model for each outcome. This meant that the random effects model for maternal commenting determined which effects were retained since there was more variability in commenting than child language outcomes. Regression effects were added to the models to examine between-dyads associations between the commenting intercepts and slopes and growth parameters for the child language outcomes. Additionally, maternal commenting residual was regressed on the residual for each child language outcome to examine within-dyad relationships between maternal commenting and child communication.

All models were estimated via maximum likelihood estimation in Mplus Version 8.4 (Muthén & Muthén, 2017). Fixed effects were tested using Wald test *p* values, whereas variance parameters were tested using likelihood ratio tests. The within-dyad model was estimated using time centered at 10 years of age. We selected this age as the center because we had data from nearly every dyad at this age and it provided a comparison point for transitioning into adolescence. Maternal commenting rate was centered at 3.5 per minute, a value near the grand mean for all mothers across all observations. Leiter IQ scores were centered at 52, close to the grand mean so that the effects of all other predictors in the model would be for someone with an average Leiter IQ score. This value was added to the model as a between-dyads predictor because of the established relationship between cognition and child communication. Pseudostandardized effects were calculated by multiplying the unstandardized coefficients by *SD*(*X*)/*SD*(*y*)—using the original standard deviation of the predictors at each level for *X* and the square root of the Level 2 random intercept or Level 1 residual variance for *Y*.

*Missing data.* Missing data are not relevant for the language outcomes and maternal commenting variables because the multivariate multilevel model uses all available data points in developing trajectories for each individual. The dyads were observed on six occasions on average, and the range was three to seven observations. Data from three participants were not included because nonverbal IQ (Leiter-R scores) was missing at their assessment near the age of 10 years. Maximum likelihood estimation will allow for missing data on outcomes, but when covariates are not available, cases are excluded.

## Results

Results are presented for each of the outcome measures for the total sample, including both boys and girls. For each outcome model, we report the growth model for commenting, the growth model for each child language variable, and how nonverbal IQ and maternal responsivity (rate of commenting) were related to child language trajectories. Differences observed in girls compared to boys are discussed after the models with the entire sample.

Prior to examining multivariate longitudinal models, we evaluated growth models for each outcome. Because commenting had the most variable trajectory and commenting was used in all the multivariate models, the random effects needed to model commenting were of specific interest. A model with random effects for commenting intercepts and slopes was compared to a model with random effects for intercepts only. Corrected chi-square tests were evaluated using the log-likelihood, scaling correction factor, and degrees of freedom for each model. The model with random intercepts and slopes fit significantly better, *X* ^2^(1) = 31.56, *p* < .001, than a model with random intercepts only. The same procedure was followed to evaluate random quadratic slopes, and their addition did not improve the model. Therefore, we modeled random intercepts and slopes for commenting and, by extension, the variables paired with commenting.

### Multivariate Model of Maternal Commenting and Rate of Different Words

This model contained 312 data points. Children averaged six observations (range: 3–7).

#### Commenting Growth

**Figure 1.**
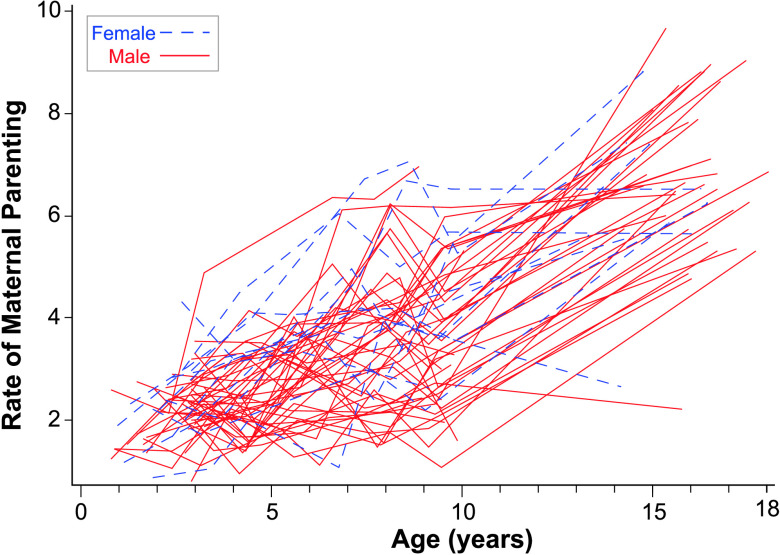
Rate of maternal commenting over time by sex. This figure plots the rate of maternal commenting per minute directed to each of the participants over all available observation points.

At age of 10 years, the fixed intercept for rate of maternal commenting was 4.21 (3.5 + 0.71) per minute. The fixed linear slope was 0.34, indicating significant growth in commenting at age of 10 years, and a significant fixed positive quadratic age slope indicating increasing slope over time. There was significant variability in intercepts, and linear slopes variance had a *p* value of .07 as indicated in Table 2 and can be observed in Figure 1. Ninety-five percent of the sample was expected to have intercepts of rate of commenting at age of 10 years between 2.22 and 6.20 per minute. Similarly, the 95% random effects confidence interval for the linear slope was between 0.13 and 0.56.

**Table 2.** Parameters for the rate of different words (RDW) model.

Different words model effects	Model 1		
	Est	*SE*	*p* <
Rate of different words model for the means			
Intercept	11.594	10.899	.287
Linear age slope (0 = 10 years)	0.303	1.205	.801
Quadratic age slope	−**0.123**	**0.012**	**.001**
Leiter IQ (0 = 52)	**0**.**118**	**0.052**	.**023**
Leiter IQ by linear	−0.003	0.010	.780
Leiter IQ by quadratic	−0.001	0.001	.128
Maternal commenting rate model for the means			
Intercept (0 = 3.5/min)	**0.714**	**0.234**	**.002**
Linear age slope (0 = 10 years)	**0.338**	**0.026**	**.001**
Quadratic age slope	**0.009**	**0.004**	**.034**
Rate of different words model for the variance			
Random intercept variance	**5.751**	**2.898**	**.047**
Linear age slope variance	0.054	0.063	.396
Intercept–age slope covariance	0.555	0.418	.184
Residual variance	**8.564**	**0.933**	**.001**
Maternal commenting rate model for the variance			
Random intercept variance	**1.032**	**0.415**	**.013**
Linear age slope variance	**0.011**	**0.006**	**.068**
Intercept–age slope covariance	**0.095**	**0.051**	**.062**
Residual variance	**0.732**	**0.123**	**.001**
Cross-variable regressions			
Commenting intercept to RDW intercept	2.925	4.529	.518
Commenting age slope to RDW age slope	−1.117	4.453	.802
Commenting intercept to RDW age slope	0.248	0.465	.594
Commenting slope to RDW intercept	−11.946	40.867	.770
Within dyad			
Commenting residual to RDW residual	**0.945**	**0.277**	**.001**
Total effect			
Commenting intercept to RDW intercept	3.871	4.553	.395
Commenting age slope to RDW age slope	−0.172	4.431	.969

*Note.* This table contains the parameter estimates (Est) for each of the effects in the model, as well as the standard error (*SE*). The probability of each effect (*p*) is presented with significant values in bold. Leiter = Leiter International Performance Scale.

#### Rate of Different Words Growth

**Figure 2.**
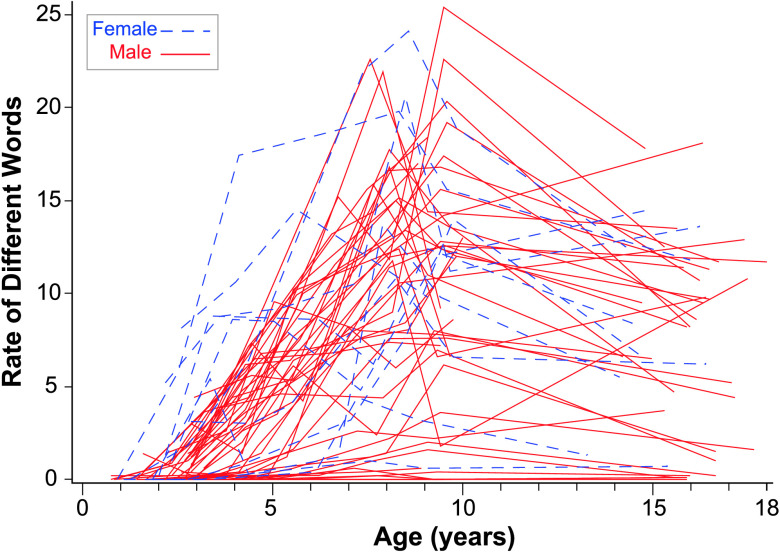
Rate of different words over time by sex. This figure plots the rate of different words per minute for each of the participants over all available observation points.

The intraclass correlation coefficient for rate of different words was .24, indicating that 24% of the variance was due to between-persons mean differences. At age of 10 years, the fixed intercept for rate of different words was 11.59 different words per minute. The fixed linear slope was 0.30 per year, which was not significantly different from zero. The fixed quadratic slope was −0.12 per year, indicating that the amount of change is significantly decreasing over time. There was significant variability in intercepts but not slopes, as shown in Figure 2. Random effects confidence intervals indicated that 95% of the sample was expected to have intercepts for rate of different words at age of 10 years between 6.89 and 16.29 per minute. Nonverbal IQ was significantly positively related to rate of different words at age of 10 years such that children's rate of different words at age of 10 years increased by 0.12 for every point increase in nonverbal IQ.

*Associations between maternal rate of commenting and child rate of different words*. The within-dyad association between commenting and rate of different words (the Level 1 effect) was significantly positive. Maternal commenting and rate of different words changed similarly over time within the dyad—for every 1-point increase in rate of commenting at a given age relative to what was predicted by one's individual trajectory, the rate of different words at that same age was expected to be higher by 0.94. The rate of commenting residual predicted rate of different words residual with a pseudostandardized effect of .28, *p* < .001.

In summary, the mothers differed in their rate of commenting at age of 10 years, and across all observations, they increasingly commented more over time on average. Similarly, the children differed in their rate of different words at age of 10 years, and while their rate of different words was not changing significantly at age of 10 years, across all of the observations, their change in rate of different words was significantly decreasing as they aged. Across the observations, mother commenting and child rate of different words tended to vary similarly. When the mother was commenting more, the child rate of different words tended to be higher.

### Multivariate Model of Maternal Commenting and MLU_m_

This model contained 209 observations because MLU_m_ was only calculated for observations where the number of utterances produced by the child was greater than or equal to 10 utterances. Only 51 of the children had an observation with 10 or more utterances and were included in this model. The average number of observations included was 4 (range: 1–7).

#### Commenting Growth

Because of the difference in data points used, the growth model for rate of maternal commenting in the MLU_m_ model is slightly different from the rate of different words model. At age of 10 years, the fixed intercept for rate of maternal commenting was 4.39 (3.5 + 0.89); the fixed linear slope was 0.33, indicating significant growth in commenting at age of 10 years, and a significant positive fixed quadratic age slope, indicating increasing positive slope over time. There was significant variability in intercepts and linear slopes across individuals, and the general shape was similar to the model with full cases. There was a positive covariance between intercept and linear age slope, indicating that mothers with higher rates of commenting at age of 10 years tended to have larger linear age slopes at age of 10 years.

#### MLU_m_ Growth

**Figure 3.**
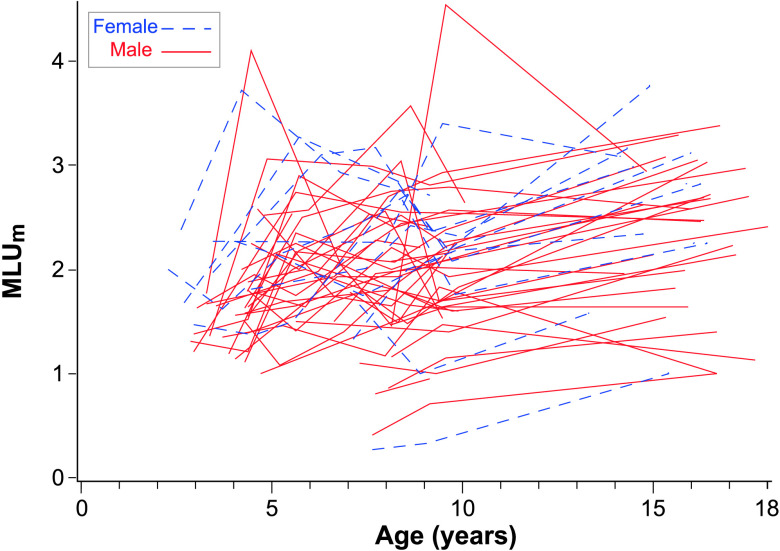
Mean length of utterance in morphemes (MLU_m_) over time by sex. This figure plots the MLU_m_ for each of the participants over all available observation points.

At age of 10 years, the fixed intercept for MLU_m_ was 1.80, and the fixed linear slope was 0.010 per year, which was not significantly different from zero. Nonverbal IQ was significantly related to intercept, but not to slope. There was significant variability in MLU_m_ intercepts at age of 10 years across participants, but not significant variability in linear age slopes. Confidence intervals indicated that 95% of the sample was expected to have intercepts for MLU_m_ at age of 10 years between 0.99 and 2.61. The trajectories for MLU_m_ can be seen in Figure 3.

*Associations between maternal rate of commenting and child MLU_m_*. Maternal rate of commenting did not predict any aspect of child MLU_m_. There were no associations between the two trajectories either within dyad or between dyads as can be seen in Table 3.

**Table 3.** Parameters for the mean length of utterance in morphemes (MLU_m_) model.

MLU_m_ model effects	Model 1		
	Est	*SE*	*p* <
MLU_m_ model for the means			
Intercept	**1.800**	**0.435**	**.001**
Linear age slope (0 = 10 years)	0.001	0.042	.980
Leiter IQ (0 = 52)	**0.021**	**0.006**	**.001**
Leiter IQ by linear	0.001	0.001	.754
Maternal commenting rate model for the means			
Intercept (0 = 3.5)	**0.890**	**0.167**	**.001**
Linear age slope (0 = 12 years)	**0.330**	**0.023**	**.001**
Quadratic age slope	**0.010**	**0.004**	**.015**
MLU_m_ model for the variance			
Random intercept variance	**0.169**	**0.051**	**.001**
Linear age slope variance	0.001	0.002	.929
Intercept–age slope covariance	0.005	0.009	.571
Residual variance	**0.191**	**0.025**	**.001**
Maternal commenting rate model for the variance			
Random intercept variance	**0.843**	**0.209**	**.001**
Linear age slope variance	**0.011**	**0.005**	**.048**
Intercept–age slope covariance	**0.048**	**0.023**	**.036**
Residual variance	**0.829**	**0.108**	**.001**
Cross-variable regressions			
Commenting intercept to MLU_m_ intercept	0.113	0.148	.447
Commenting age slope to MLU_m_ age slope	0.165	0.157	.291
Commenting intercept to MLU_m_ age slope	0.012	0.016	.464
Commenting slope to MLU_m_ intercept	0.506	1.534	.742
Within dyad			
Commenting residual to MLU_m_ residual	−0.010	0.039	.798
Total effect			
Commenting intercept to MLU_m_ intercept	0.103	0.141	.467
Commenting age slope to MLU_m_ age slope	0.155	0.149	.297

*Note.* This table contains the parameter estimates (Est) for each of the effects in the model, as well as the standard error (*SE*). The probability of each effect (*p*) is presented with significant values in bold. Leiter = Leiter International Performance Scale.

In summary, in this model, mothers continued to differ in their rate of commenting at age of 10 years and in the amount that they increased their commenting over time. The average rate of commenting when the children were 10 years of age was 4.39 comments per minute, and child MLU_m_ at that age averaged 1.80. Ten-year-old children with higher nonverbal IQs tended to have higher MLU_m_ than children with lower IQs. Maternal commenting was not related to changes in MLU_m_.

### Multivariate Model for Maternal Commenting and PPVT-4

The PPVT-4 was added to the battery of tests part way through the longitudinal study. Therefore, the number of data points for this model was 153. Fifty-three children contributed an average of 3 data points to this model (range: 1–4).

#### Commenting Growth

Because of the fewer data points used, the growth model for rate of maternal commenting in the PPVT-4 model only includes linear growth (i.e., quadratic growth was not modeled). At age of 10 years, the fixed intercept for rate of maternal commenting was 4.29 (3.5 + 0.79); the fixed linear slope was 0.37, indicating significant growth in commenting over time. There is significant variability in intercepts for commenting, but not in linear slopes.

#### PPVT-4 Growth

**Figure 4.**
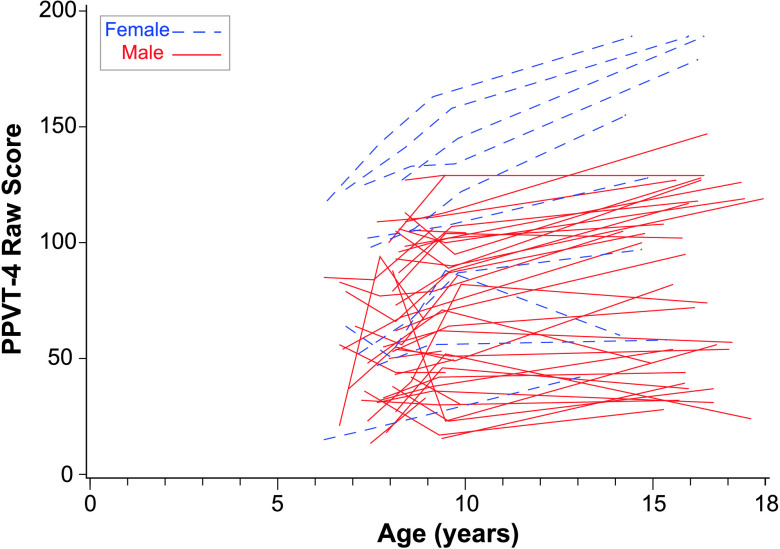
Peabody Picture Vocabulary Test–Fourth Edition (PPVT-4) over time by sex. This figure plots the raw score from the PPVT-4 for each of the participants over all available observation points.

At age of 10 years, the fixed intercept for PPVT-4 raw score was 69.53, and the fixed linear slope was significant with an increase of 2.12 words per year. There was significant variability in PPVT-4 intercepts at age of 10 years across participants but not significant variability in linear age slopes. Random effects confidence intervals indicated that 95% of the sample is expected to have intercepts for PPVT-4 at age of 10 years between 34.50 and 104.56. The trajectories for PPVT-4 can be seen in Figure 4. Nonverbal IQ was significantly related to intercepts and slopes such that individuals' PPVT-4 score at age of 10 years increased by 2.11 and slope increased by 0.09 for every Leiter IQ point.

*Associations between maternal rate of commenting and PPVT-4*. Maternal commenting intercept was predictive of PPVT-4 linear slope between dyads. In other words, moms who commented more at age of 10 years than other moms have children who have larger increases in PPVT-4 scores over time. There were no other significant associations between maternal commenting and PPVT-4 scores either within dyad or between dyads, as can be seen in Table 4.

**Table 4.** Parameters for the Peabody Picture Vocabulary Test–Fourth Edition (PPVT-4) model.

PPVT-4 raw score model effects	Model 1		
	Est	*SE*	*p* <
PPVT-4 model for the means			
Intercept	**69.529**	**14.025**	**.001**
Linear age slope (0 = 10 years)	**2.120**	**0.696**	**.002**
Leiter IQ (0 = 52)	**2.105**	**0.180**	**.001**
Leiter IQ by linear	**0.094**	**0.025**	**.001**
Maternal commenting rate model for the means			
Intercept (0 = 3.5)	**0.804**	**0.171**	**.001**
Linear age slope (0 = 12 years)	**0.367**	**0.031**	**.001**
PPVT-4 model for the variance			
Random intercept variance	**319.402**	**76.261**	**.001**
Linear age slope variance	0.195	4.390	.965
Intercept–age slope covariance	6.914	7.641	.366
Residual variance	**146.502**	**33.717**	**.001**
Maternal commenting rate model for the variance			
Random intercept variance	**1.201**	**0.242**	**.001**
Linear age slope variance	**0.367**	**0.031**	**.001**
Intercept–age slope covariance	−0.021	0.037	.577
Residual variance	**0.867**	**0.115**	**.001**
Cross-variable regressions			
Commenting intercept to PPVT-4 intercept	2.691	2.832	.342
Commenting age slope to PPVT-4 age slope	−0.787	2.402	.743
Commenting intercept to PPVT-4 age slope	**0.942**	**0.281**	**.001**
Commenting slope to PPVT-4 intercept	18.408	38.795	.635
Within dyad			
Commenting residual to PPVT-4 residual	0.239	1.107	.829
Total effect			
Commenting intercept to PPVT-4 intercept	2.930	2.622	.264
Commenting age slope to PPVT-4 age slope	−0.548	1.785	.759

*Note.* This table contains the parameter estimates (Est) for each of the effects in the model, as well as the standard error (*SE*). The probability of each effect (*p*) is presented with significant values in bold. Leiter = Leiter International Performance Scale.

In summary, moms differed in their rate of commenting at age of 10 years and in how they changed in their rate of commenting as the child aged. On average, rate of commenting increased over time. Similarly, children differed in their PPVT scores at 10 years of age. Scores increased significantly over time, on average. There were no significant differences between children in how much their PPVT scores were changing over time. Children with higher IQs at age of 10 years tended to also have higher PPVT scores at age of 10 years and greater increases over time than children with lower IQs.

### Multivariate Model for Maternal Commenting and EVT-2

The EVT-2 was also added to the battery later, and because some children did not communicate symbolically, they did not have scores to include in the model. Therefore, this analysis included 138 observations across 47 children. The average number of observations was 3, with a range of 1–4.

#### Commenting Growth

Because of the fewer data points used, the growth model for rate of maternal commenting in the EVT-2 model only includes linear growth. At age of 10 years, the fixed intercept for rate of maternal commenting was 4.48 (3.5 + 0.98); the fixed linear slope was 0.37, indicating significant growth in commenting over time. There is significant variability in intercepts for commenting, but not in linear slopes.

#### EVT-2 Growth

**Figure 5.**
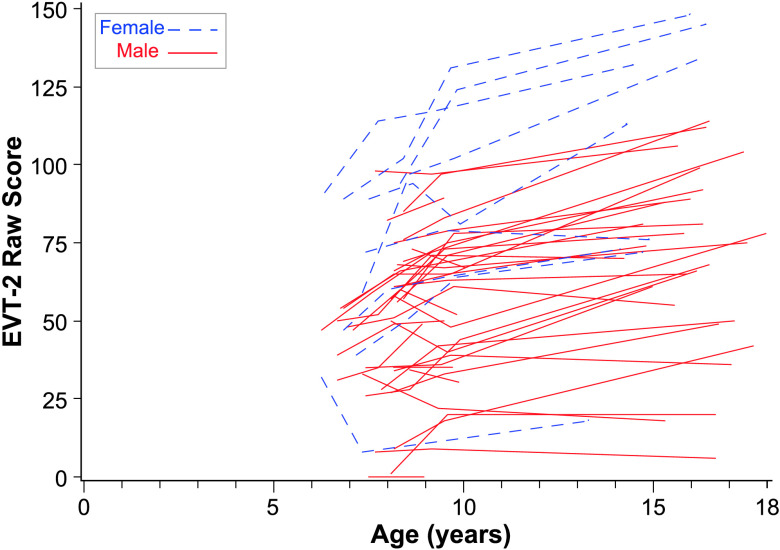
Expressive Vocabulary Test–Second Edition (EVT-2) over time by sex. This figure plots the raw score from the EVT-2 for each of the participants over all available observation points.

Because most children did not have 4 data points, we only modeled linear growth in both EVT and maternal commenting. At age of 10 years, the fixed intercept for EVT-2 raw score was 50.23, and the fixed linear slope was not significant. There was significant variability in EVT-2 intercepts at age of 10 years across participants, but not significant variability in linear age slopes. Random effects confidence intervals indicated that 95% of the sample is expected to have intercepts for EVT-2 at age of 10 years between 23.45 and 77.01. The trajectories for EVT-2 can be seen in Figure 5. Leiter IQ was significantly related to intercept such that individuals' EVT-2 score at age of 10 years increased by 1.69 for every IQ point increase. Leiter IQ also significantly influenced slope, with slopes increasing by 0.08 for every point of IQ increase.

*Associations between maternal rate of commenting and child EVT-2*. There were no significant associations between maternal commenting and EVT scores, as can be seen in Table 5.

**Table 5.** Parameters for the Expressive Vocabulary Test–Second Edition (EVT-2) model.

EVT-2 raw score model effects	Model 1		
	Est	*SE*	*p* <
EVT-2 model for the means			
Intercept	50.234	28.703	.080
Linear age slope (0 = 10 years)	2.549	1.931	.187
Leiter IQ (0 = 52)	**1.691**	**0.367**	**.001**
Leiter IQ by linear	**0.076**	**0.038**	**.042**
Maternal commenting rate model for the means			
Intercept (0 = 3.5)	**0.973**	**0.286**	**.001**
Linear age slope (0 = 12 years)	**0.380**	**0.060**	**.001**
EVT-2 model for the variance			
Random intercept variance	**186.644**	**57.931**	**.001**
Linear age slope variance	0.164	1.480	.912
Intercept–age slope covariance	2.535	6.331	.689
Residual variance	**53.007**	**23.007**	**.021**
Maternal commenting rate model for the variance			
Random intercept variance	1.022	0.546	.061
Linear age slope variance	0.018	0.014	.196
Intercept–age slope covariance	−0.048	0.102	.639
Residual variance	**0.939**	**0.370**	**.011**
Cross-variable regressions			
Commenting intercept to EVT-2 intercept	0.912	6.525	.889
Commenting age slope to EVT-2 age slope	−1.170	4.683	.706
Commenting intercept to EVT-2 age slope	0.211	0.470	.654
Commenting slope to EVT-2 intercept	17.399	60.712	.774
Within dyad			
Commenting residual to EVT-2 residual	1.211	1.549	.435
Total effect			
Commenting intercept to EVT-2 intercept	2.123	5.953	.721
Commenting age slope to EVT-2 age slope	−0.559	4.207	.894

*Note.* This table contains the parameter estimates (Est) for each of the effects in the model, as well as the standard error (*SE*). The probability of each effect (*p*) is presented with significant values in bold. Leiter = Leiter International Performance Scale.

In summary, with this reduced sample, mothers differed in their rate of commenting at age of 10 years, but not in the extent to which their rate of commenting was increasing over time. Children differed in their expressive vocabulary at age of 10 years, but EVT-2 scores did not change significantly over time. At age of 10 years, children with higher IQs tended to have higher expressive vocabulary. Children with higher IQs at age of 10 years tended to have greater increases in expressive vocabulary over time.

### Influence of Sex on Trajectories

While we did not have enough females in our sample to model trajectories for males and females separately, the figures for each language variable denote the sex for each child participant. There are no consistent advantages for females in the sample except for the PPVT-4 and the EVT-2, where the top four to five trajectories were girls. While at the early ages, there were several females at the top of the distribution in terms of rate of different words, by the middle childhood ages, the girls were more evenly distributed across the top, middle, and bottom of the distribution.

In terms of maternal commenting, there may be more commenting to girls in early adolescence in the rate of different words model (see Figure 1). Several of the top trajectories were from mothers commenting to girls, and only one mother of a girl was in the bottom portion of the distribution. However, there was substantial overlap in commenting distributions across boys and girls.

To gain more information about potential sex differences, the models were rerun excluding females (see Supplemental Material S1). A comparison of the models for the total sample to the models for boys only revealed very similar growth parameters overall across the samples. However, we observed three sex-related differences: (a) relationships with the Leiter, (b) intercept differences, and (c) relationships among growth parameters. Leiter IQ scores were related to PPVT-4 and EVT-2 linear age slopes in the total sample model but not the boys-only model. Thus, the relationship between cognition and these different types of outcomes may have differential sex effects that need further exploration. Second, the rate of maternal commenting at age of 10 years was higher in the total sample than in the boys-only sample. Thus, maternal commenting occurred with higher frequency to daughters than to sons. Rate of different words at age of 10 years was also notably higher in the total sample model (11.59) than in the boys-only model (7.98). Finally, the relationships between growth parameters were sometimes stronger in the total sample model than the boys-only model. For example, the commenting intercept was related to PPVT-4 linear slope in the total sample but not in the boys-only sample, suggesting that this effect is somewhat stronger for the females in the sample than for the males.

## Discussion

Overall, these findings indicate that, while language continues to be very delayed for most participants, we nevertheless detected continued growth in several aspects of language in adolescents with FXS. Positive slopes were detected for vocabulary measured by standardized tests and in the language sample. Rates of number of different words grew steadily for most participants until age of 10 years and then decreased somewhat. However, several participants continued to produce or understand very few words, as can be seen in Figures 2, 3, 4, and 5.

Mean length of utterance in morphemes did not change significantly from the low intercept values determined at age of 10 years, however. While we did not examine grammaticality of productions in more detail, our results suggest that grammar and syntax do not appear to be significantly changing between the ages of middle childhood and adolescence because the length of utterances is not changing. These results are commensurate with single time point studies of adolescents with FXS that found deficits in syntax relative to their cognitive-matched TD peers (Finestack & Abbeduto, 2010; Kover et al., 2012). Low skills in grammar and syntax may impair adolescents' abilities to socially communicate and participate in academic language tasks (Abbeduto et al., 2019; Haebig et al., 2016). McDuffie et al. (2018) found that adolescents who participated in an intervention aimed at improving lexical diversity and grammatical complexity increased their lexical diversity; however, grammatical complexity did not change. These findings are further indication that grammaticality is particularly impaired and difficult to treat in children and adolescents with FXS.

### Maternal Responsivity

We further investigated the role of maternal responsivity on language growth by examining how language outcomes changed in relation to maternal commenting. We examined commenting as a predictor of each language outcome. Two vocabulary outcomes were predicted by maternal commenting—rate of different words and PPVT-4 scores. These outcomes extend previous findings that responsivity was positively related to growth in number of different words through middle childhood (Brady et al., 2014). Results from our analyses indicate that changes in maternal responsivity and number of different words covary. That is, when mothers commented more, their children had higher rates of different words and vice versa. Children whose mothers commented more at age of 10 years also showed greater increases in receptive vocabulary as measured with the PPVT-4. However, maternal commenting rates were not predictive of intercepts or growth in MLU_m_ or EVT-2 raw scores. The MLU_m_ finding is similar to what we found in a previous study, which also showed that MLU variability was unrelated to maternal responsivity (Komesidou et al., 2017). Apart from growth in EVT-2 raw scores, it appears that maternal responsivity is more related to vocabulary development than to syntax.

Our results illustrate the advantages of using multivariate multilevel models when examining dyadic interactions. We included time-varying covariates so that we could see how changes in maternal commenting over time related to changes in trajectories of language variables. The use of multivariate multilevel models allowed us to simultaneously estimate the trajectories for our child outcomes of interest and our maternal commenting predictor. Adding the regression effects enabled us to examine the multiple ways in which the trajectories of maternal commenting could predict trajectories of child outcomes—within dyad for the residuals and between dyads for the intercepts and slopes. This approach, which separately examines the within-dyad and between-dyads effects, enabled us to see that, while the effect of commenting between dyads on the intercept and slope for rate of different words was not significant, the within-dyad effect was significant. This finding illustrates how important it is to appropriately partition the variance into the between and within components, so that between-dyads effects are not confounded with within-dyad effects. Finally, this approach emphasizes the importance of studying individual differences in developmental research.

### Clinical Implications

The findings from this longitudinal study, extending into adolescence, have several clinical implications for older children with FXS. First, having a responsive mother as a communication partner continues to have a positive impact on vocabulary skills during adolescence. This suggests that interventions targeting responsive interaction, with a clear emphasis on commenting, by a variety of communication partners, including nonaffected siblings, TD peers, and teachers, are likely to provide continued benefit for vocabulary growth through adolescence.

A second result with clinical implications is the lack of growth in MLU_m_ and lack of effect of maternal responsivity on MLU_m_. We expected to see increased MLU_m_ as children grew older and developed more advanced vocabulary skills. However, this was not the case for many participants in our study. The use of more complex syntax relates to both academic success and employment outcomes (Power-deFur, 2015) and should be prioritized. Our findings are commensurate with those of McDuffie et al. (2018), who found that increasing maternal responsivity did not result in improved syntax. Together, these findings suggest the need for research on more direct approaches to improve syntax. For example, teaching youths with FXS to use conjunctions to combine simple sentences and to embed clauses to produce complex sentences may lead to longer more complex sentences. Such interventions may also benefit receptive syntax. Although the current study focused on expressive outcomes, Oakes et al. (2013) found that receptive syntax was also lower than expected in youths with FXS.

### Role of Sex

Our analyses included males and females with FXS. We consider this a strength because many previous studies have excluded females, and less is known about their language development. Girls in our study presented a range of language abilities, similar to girl participants described by Sterling and Abbeduto (2012). Differences observed between the boys-only and total samples indicate that the greater variability introduced by girls was responsible for some of our results. In addition, the differences in commenting to girls versus boys suggest that maternal input may contribute to differences observed in girls over time.

Our findings can be compared to previous studies of groups that contained males and females. Pierpont et al. (2011) measured receptive and expressive vocabulary and syntax in a group of adolescent boys and girls with FXS at two time points. Girls had higher mean scores at the first time point (12 years of age) and made more changes over the next 2 years on measures of vocabulary and expressive syntax. Similar to our findings, however, there was wide variation for all language measures and overlap in distributions for both boys and girls. Finestack and Abbeduto (2010) compared expressive language measured with standardized tests and language sample analyses in five female adolescents to 12 male adolescents. Mean scores for girls were higher than for boys, but the scores from the language sample indicated that there was substantial variability within and across groups of boys and girls. Komesidou et al. (2017) reported higher scores in syntax as measured with the IPSyn (Scarborough, 1990) and MLU over time for girls than boys, but there were many girls whose scores on both measures fell within the distribution of boys (Komesidou et al., 2017).

Interestingly, advantages for girls were most obvious on the standardized vocabulary tests—PPVT-4 and EVT-2. It may be that some of the reported advantages for girls reflect superior test-taking abilities rather than advantages observed during conversation. It should be noted that the girls were still performing lower than age expectations and many were performing at low levels commensurate with boys. Thus, for all boys and many girls, expressive language, particularly during conversational interaction, remains a need to address in intervention. Impaired language is likely to impact not only social interactions but also communication in postsecondary education and vocational experiences. Further examination of girls with FXS is warranted.

### Strengths and Limitations

In the current study, we were able to extend our longitudinal study from early childhood into adolescence. A strength is the retention and stability of the sample over time and our collection of multiple data points across multiple observations. However, we faced challenges associated with data collection across developmental periods. Our study has included naturalistic observations of mother–child interactions across time. In our attempt to obtain representative samples that are age appropriate, we adapted several contexts over time. In our current study, we identified variability in outcomes that were associated with these contextual differences, thus necessitating elimination of some contexts and instead focusing on one context (snack) for comparison over time. Use of snack may limit the type of vocabulary used, and longer samples from more diverse contexts are recommended for future studies. In addition, nonverbal IQ, as assessed by the Leiter, was only available at later observation periods because a different measure (i.e., Mullen Scales of Early Learning) was used during the first three visits when the children were very young.

Another limitation in the current study is a lack of emphasis on pragmatic aspects of language, including the use of perseverations. The use of perseverative language by children and adolescents with FXS has been described as a major concern, possibly related to syntactic deficits (Martin et al., 2012; Sudhalter et al., 1992). Impaired communicative repair strategies have also been described in boys with FXS (Abbeduto et al., 2008; Fielding-Gebhardt et al., 2020). Our most recent data include information about perseverations and other aspects of pragmatic language, but these variables were not available from earlier time points. A summary of pragmatic language variables observed during adolescence is in preparation.

We included girls and boys in our analyses. This is important because there is little information currently available about development in girls or adolescents with FXS. However, we did not have enough girls in our sample to examine their results separately or to compare their results to boys statistically. Instead, we provided visual and descriptive results to allow readers to consider how girls performed relative to boys in our sample. Models with boys only are provided in Supplemental Material S1.

### Future Directions

Further examination of language strengths and weaknesses within the adolescent period is needed. This is a need across all individuals with intellectual disabilities, including those with FXS. Results obtained from the adolescent period not only indicate how development compares to earlier ages (as was the focus of the current study) but also set the stage for transitions into adulthood. Communication abilities are key variables in terms of future outcomes in adulthood, including living environments, quality of life, employment options, and personal relationships. Further research is needed to identify and treat communication skills that are amenable to intervention beyond the childhood years so that adults with FXS may fully maximize their ability to participate in society.

## Conclusions

Adolescents with FXS continue to face significant challenges in terms of communication. Developmental trajectories from early childhood show continued growth in expressive and receptive vocabulary, although levels remain much lower than in typical development. Less growth was observed in MLU_m_, indicating that syntax is even more impaired than vocabulary. Although generally less effected than boys, girls showed similar patterns of language growth. Maternal responsivity continues to be significantly related to vocabulary growth, after accounting for differences in nonverbal IQ. These findings support intervention efforts aimed at continuing to provide facilitative language input through adolescence. However, our findings indicate that responsivity effects are not sufficient to impact syntax and suggest the need for more focused interventions aimed at helping children with FXS produce more complex sentences.

## Supplementary Material

10.1044/2020_JSLHR-20-00198SMS1 Supplemental Material S1 Models rerun excluding females.
