# Supplementary material for: Language Development From Early Childhood to Adolescence in Youths With Fragile X Syndrome
Source: J Speech Lang Hear Res. 2020 Oct 1;63(11):3727–42. doi: 10.1044/2020_JSLHR-20-00198 (PMC8582761; doi:10.1044/2020_JSLHR-20-00198)
Supplement: Supplemental Material S1 [file JSLHR-63-3727-s001.pdf]

Supplemental Material S1.

Table S1.
Parameters for the Rate of Different Words Model for Males Only

Different Words Model Effects	Model 1		
	Est	*SE*	*p* <
Rate of Different Words Model for the Means			
Intercept	**7.978**	**18.476**	**.666**
Linear Age Slope (0 = 10 years)	.363	3.234	.911
Quadratic Age Slope	**-.145**	**.016**	**.001**
Leiter IQ (0 = 52)	**.269**	**.095**	**.005**
LeiterIQ by Linear	.015	.018	.415
LeiterIQ by Quadratic	-.003	.002	.180
Maternal Commenting Rate Model for the Means			
Intercept (0 = 3.5/min)	.569	.350	.104
Linear Age Slope (0 = 10 years)	**.353**	**.050**	**.001**
Quadratic Age Slope	**.013**	**.005**	**.006**
Rate of Different Words Model for the Variance			
Random Intercept Variance	5.631	3.629	.121
Linear Age Slope Variance	.059	.074	.425
Intercept-Age Slope Covariance	.575	.399	.149
Residual Variance	**7.452**	**.956**	**.001**
Maternal Commenting Rate Model for the Variance			
Random Intercept Variance	**.971**	**.470**	**.039**
Linear Age Slope Variance	.013	.008	.136
Intercept-Age Slope Covariance	.106	.065	.104
Residual Variance	**.692**	**.136**	**.001**
Cross-Variable Regressions –			
Commenting intercept to RDW Intercept	.426	6.976	.951
Commenting age slope to RDW age slope	6.771	63.343	.915
Commenting intercept to RDW age slope	.139	1.237	.911
Commenting slope to RDW intercept	6.771	63.343	.915
Within-Dyad			
Commenting residual to RDW residual	**1.118**	**.480**	**.020**
Total Effect			
Commenting intercept to RDW Intercept	1.544	7.169	.829
Commenting age slope to RDW age slope	.173	11.232	.988

Note. This table contains the parameter estimates (Est) for each of the effects in the model as well as the standard error (*SE*).
The probability of each effect (*p*) is presented with significant values in bold.

Table S2.
Parameters for the MLU-m Model for Males Only

MLU Model Effects	Model 1		
	Est	*SE*	*p* <
<u>MLU Model for the Means</u>			
Intercept	**1.810**	**1.003**	**.071**
Linear Age Slope (0 = 10 years)	.010	.075	.895
Leiter IQ (0 = 52)	**.023**	**.009**	**.014**
LeiterIQ by Linear	.001	.001	.440
<u>Maternal Commenting Rate Model for the Means</u>			
Intercept (0 = 3.5)	**.806**	**.182**	**.001**
Linear Age Slope (0 = 12 years)	**.355**	**.025**	**.001**
Quadratic Age Slope	**.012**	**.005**	**.016**
<u>MLU Model for the Variance</u>			
Random Intercept Variance	**.136**	**.046**	**.003**
Linear Age Slope Variance	.001	.004	.962
Intercept-Age Slope Covariance	.005	.008	.556
Residual Variance	**.188**	**.025**	**.001**
<u>Maternal Commenting Rate Model for the Variance</u>			
Random Intercept Variance	**.710**	**.244**	**.004**
Linear Age Slope Variance	.008	.005	.129
Intercept-Age Slope Covariance	**.057**	**.031**	**.066**
Residual Variance	**.845**	**.132**	**.001**
<u>Cross-Variable Regressions –</u>			
Commenting intercept to MLU Intercept	.202	.327	.536
Commenting age slope to MLU age slope	.110	.266	.678
Commenting intercept to MLU age slope	.022	.028	.425
Commenting slope to MLU intercept	.359	3.482	.918
Within-Dyad			
Commenting residual to MLU residual	-.011	.041	.779
Total Effect			
Commenting intercept to MLU Intercept	.191	.318	.548
Commenting age slope to MLU age slope	.099	.259	.702

Note. This table contains the parameter estimates (Est) for each of the effects in the model as well as the standard error (*SE*).
The probability of each effect (*p*) is presented with significant values in bold.

Table S3.
Parameters for the PPVT-IV Model for Males Only

PPVT-4 Raw Score Model Effects	Model 1		
	Est	*SE*	*p* <
<u>PPVT-4 Model for the Means</u>			
Intercept	**72.436**	**26.177**	**.006**
Linear Age Slope (0 = 10 years)	2.073	4.823	.667
Leiter IQ (0 = 52)	**2.415**	**.905**	**.008**
LeiterIQ by Linear	.037	.086	.667
<u>Maternal Commenting Rate Model for the Means</u>			
Intercept (0 = 3.5)	**.703**	**.370**	**.058**
Linear Age Slope (0 = 12 years)	**.392**	**.073**	**.001**
<u>PPVT-4 Model for the Variance</u>			
Random Intercept Variance	**341.127**	**179.807**	**.058**
Linear Age Slope Variance	.255	6.740	.970
Intercept-Age Slope Covariance	8.435	17.059	.621
Residual Variance	**161.134**	**36.241**	**.001**
<u>Maternal Commenting Rate Model for the Variance</u>			
Random Intercept Variance	1.189	.841	.158
Linear Age Slope Variance	.019	.012	.118
Intercept-Age Slope Covariance	-.001	.068	.990
Residual Variance	**.759**	**.326**	**.020**
<u>Cross-Variable Regressions –</u>			
Commenting intercept to PPVT-4 Intercept	1.560	8.932	.861
Commenting age slope to PPVT-4 age slope	-1.739	15.150	.909
Commenting intercept to PPVT-4 age slope	1.089	1.509	.471
Commenting slope to PPVT-4 intercept	17.449	60.790	.774
Within-Dyad			
Commenting residual to PPVT-4 residual	.262	3.179	.934
Total Effect			
Commenting intercept to PPVT-4 Intercept	1.822	7.230	.801
Commenting age slope to PPVT-4 age slope	-1.477	13.645	.914

Note. This table contains the parameter estimates (Est) for each of the effects in the model as well as the standard error (*SE*).
The probability of each effect (*p*) is presented with significant values in bold.

Table S4.
EVT-2 Model Parameters for Males Only

EVT-2 Raw Score Model Effects	Model 1		
	Est	*SE*	*p* <
<u>EVT-2 Model for the Means</u>			
Intercept	47.845	39.840	.230
Linear Age Slope (0 = 10 years)	1.101	2.963	.710
Leiter IQ (0 = 52)	**2.045**	**.606**	**.001**
LeiterIQ by Linear	.042	.067	.535
<u>Maternal Commenting Rate Model for the Means</u>			
Intercept (0 = 3.5)	**.880**	**.369**	**.017**
Linear Age Slope (0 = 12 years)	**.412**	**.093**	**.001**
<u>EVT Model for the Variance</u>			
Random Intercept Variance	**209.653**	**103.757**	**.043**
Linear Age Slope Variance	.270	1.733	.876
Intercept-Age Slope Covariance	5.122	8.443	.544
Residual Variance	**34.682**	**15.824**	**.028**
<u>Maternal Commenting Rate Model for the Variance</u>			
Random Intercept Variance	.988	.618	.110
Linear Age Slope Variance	.015	.019	.442
Intercept-Age Slope Covariance	-.036	.134	.790
Residual Variance	**.851**	**.382**	**.026**
<u>Cross-Variable Regressions –</u>			
Commenting intercept to EVT-2 Intercept	1.624	8.886	.855
Commenting age slope to EVT-2 age slope	1.842	6.964	.791
Commenting intercept to EVT-2 age slope	.251	.667	.706
Commenting slope to EVT-2 intercept	26.366	81.066	.745
Within-Dyad			
Commenting residual to EVT-2 residual	.830	1.491	.578
Total Effect			
Commenting intercept to EVT-2 Intercept	2.454	8.988	.785
Commenting age slope to EVT-2 age slope	2.671	6.363	.675

Note. This table contains the parameter estimates (Est) for each of the effects in the model as well as the standard error (*SE*).
The probability of each effect (*p*) is presented with significant values in bold.
